# Hepatitis C Virus and Human Cytomegalovirus—Natural Killer Cell Subsets in Persistent Viral Infections

**DOI:** 10.3389/fimmu.2017.00566

**Published:** 2017-05-17

**Authors:** Julia Pollmann, Alexander Rölle, Maike Hofmann, Adelheid Cerwenka

**Affiliations:** ^1^Research Group Innate Immunity, German Cancer Research Center (DKFZ), Heidelberg, Germany; ^2^Clinical Cooperation Unit Applied Tumor-Immunity, German Cancer Research Center (DKFZ), National Center for Tumor Diseases (NCT), Heidelberg, Germany; ^3^Faculty of Medicine, Department of Medicine II, University Hospital Freiburg, University of Freiburg, Freiburg im Breisgau, Germany; ^4^Medical Faculty Mannheim, Division of Immunbiochemistry, University Heidelberg, Heidelberg, Germany

**Keywords:** natural killer cells, hepatitis C virus, human cytomegalovirus, chronic infection, natural killer subsets

## Abstract

Hepatitis C virus (HCV) and human cytomegalovirus (HCMV) are prominent examples of RNA and DNA viruses, respectively, that establish a persistent infection in their host. HCV affects over 185 million patients worldwide, who are at high risk for developing liver fibrosis, liver cirrhosis, and ultimately hepatocellular carcinoma. Recent breakthroughs in HCV therapy, using direct-acting antivirals have provided the opportunity to monitor natural killer (NK) cells after clearance of a chronic infection. There is now increasing evidence that the individual NK cell repertoire before infection is predictive for the course of disease. HCMV affects the majority of the global population. While being asymptomatic in healthy individuals, HCMV represents a severe clinical challenge in immunocompromised patients. Both viral infections, HCV and HCMV, lead to long-lasting and profound alterations within the entire NK cell compartment. This review article, will discuss the diverse range of changes in the NK cell compartment as well as potential consequences for the course of disease.

## Introduction

A wide range of viral infections challenge the immune system throughout the lifetime of its host exerting a substantial and often long-lasting impact on multiple immune parameters. Natural killer (NK) cells, vital players in the antiviral immune defense, have been shown to undergo substantial changes in phenotype, function, and subset distribution during persistent viral infections. Specific NK subsets have been associated with both efficient clearance of viruses and immune dysfunction.

Persistent viral infections can be latent or chronic. Latent infection is characterized by long periods of viral inactivity with no replication or production of new virions although stress stimuli can trigger episodes of reactivations. Prominent examples of viruses establishing latent infection are the herpes viruses [human cytomegalovirus (HCMV), herpes simplex virus (HSV), Epstein–Barr virus (EBV), varicella-zoster-virus (VZV)]. Other viruses, such as the majority of hepatitis viruses [hepatitis C Virus (HCV), hepatitis B Virus (HBV), hepatitis D Virus (HDV)] and human immunodeficiency virus (HIV), establish chronic infections in which constant replication takes place. This drives chronic inflammation, often resulting in severe tissue damage of the infected organ (1). In this review, we will focus primarily on the effects of latent HCMV and chronic HCV infection on NK cells.

Hepatitis C Virus is a hepatotropic, enveloped, (+)-strand RNA virus that is transmitted person-to-person *via* blood and establishes chronic infection in 55–85% of patients. The probability for spontaneous viral clearance depends on several factors such as age, sex, host genetic factors, coinfection with other viruses, and viral genotype (2, 3).

Currently, seven genotypes and multiple subgenotypes are described with distinct global distribution patterns. In developed countries, genotype 1 is the most common, accounting for around 50% of all HCV infections, even though it has the most favorable prognosis (4). Worldwide an estimated 2.5% of the world’s population is chronically infected with HCV. Throughout the decade-long infection, the liver suffers from immunopathology, resulting in fibrosis, cirrhosis, often progressing to hepatocellular carcinoma. Each year around 500,000 people die from HCV-related liver diseases (5). To establish chronicity, the virus interferes with several innate and adaptive immune pathways, such as recognition by retinoic acid inducible gene I (RIG-I), the primary sensor for HCV-RNA in the host cell’s cytoplasm (6). The emergence of viral escape variants facilitates evasion from recognition by CD8 T cells, which are the main effector cells against HCV (7).

Until 2011, standard therapy for HCV consisted of pegylated IFN-α/ribavirin. However, only around half of the patients achieved a sustained virological response (SVR) defined by no detectable HCV-RNA 24 weeks after treatment and side effects were drastic. In 2011, the first direct-acting antivirals (DAAs) were approved targeting essential viral proteins which revolutionized therapy by reaching SVR rates of >90% (4, 8, 9).

Human cytomegalovirus has a linear double-stranded DNA genome of 236 kbp. The virus spreads vertically and horizontally *via* bodily fluids by infecting epithelial and endothelial cells, macrophages, and DCs wherein it establishes life-long latency. This leads to high global prevalence rates of 60–85%, depending on socioeconomic factors, geographical location, and age. HCMV is an opportunistic pathogen, causing disease only in immunocompromised people, e.g., during transplantations or in HIV patients. Furthermore, transplacental transmission of HCMV can cause severe, primarily neurological, damage to the fetus (10).

Human cytomegalovirus has developed a plethora of strategies and dedicates a large portion of its genome to interfere with the host immune system. Many of these escape mechanisms have evolved to avoid recognition by NK cells (11–13).

Natural killer cells are important effector cells in the antiviral immune response in during HCMV and HCV infection (14–17).

The importance of NK cells in human Herpes virus infections was initially highlighted in a patient with a very rare NK cell deficiency and his enhanced susceptibility to recurrent infections (18), a clinical phenotype corroborated in later reports (19, 20). Moreover, it is also indirectly evident in the multiple immune evasion strategies that HCMV utilizes to prevent NK cell recognition (11, 12).

**Figure 1:**
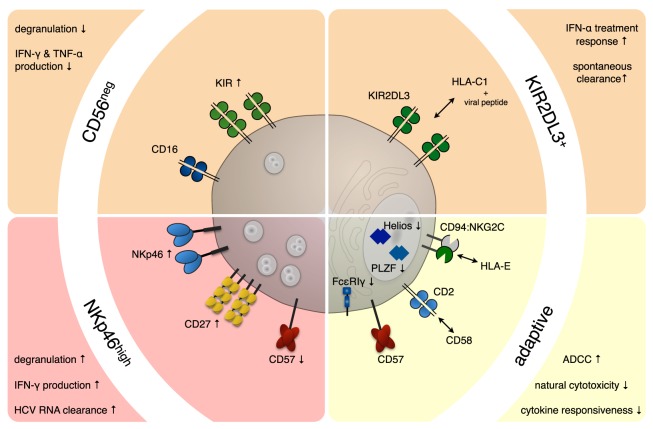
**NK cell subsets in chronic viral infections**. Persistent infections exert a profound and long-lasting impact on the NK cell compartment. In hepatitis C virus (HCV) (red background) and human cytomegalovirus (HCMV) (yellow background) infection, a number of NK cell subsets have been shown to expand and/or correlate with disease progression and outcome. During chronic HCV infection, an NK cell subset displaying increased expression of the natural cytotoxicity receptor NKp46 was observed, which proved superior in clearing HCV-RNA from infected hepatocytes. On the other hand, expansions of functionally impaired CD56neg NK cells (CD56^−^CD16^+^CD3^−^CD14^−^CD19^−^) were described during chronic HCV and human immunodeficiency virus and sporadically during reactivation episodes of HCMV. HCV patients with high numbers of NK cells expressing the inhibitory receptor KIR2DL3 on an HLA-C1/C1 background are more likely to spontaneously clear the virus or respond favorably to IFN-α treatment. Furthermore, certain killer immunoglobulin-like receptors (KIRs), including KIR2DL3 seem to be overrepresented on expanded adaptive NK cell subsets in HCMV infection. These adaptive NK cell subsets often express NKG2C and CD57 and are characterized by low/absent levels of key signaling molecules (e.g., FcεRIγ) and transcription factors (e.g., PLZF and/or Helios).

The two main strategies employed by various viruses to evade NK cells are preventing the upregulation of activating ligands or enhancing the expression of ligands for inhibitory NK cell receptors. Upon viral infection, a variety of stress-induced molecules are expressed on the surface of infected cells, which can be recognized by activating receptors on NK cells. Important activating receptors belong to the natural cytotoxicity receptor (NCR) family, including NKp30, NKp44, and NKp46, which can recognize cellular as well as viral ligands. However, many of the NCR ligands still remain elusive. MHC class I molecules, in particular HLA-C, provide the main inhibitory signals for NK cells *via* interacting with killer immunoglobulin-like receptors (KIRs) (21). The KIRs, like their MHC ligands, are genetically highly polymorphic and expressed in a stochastic manner, leaving every NK cell with 0–4 KIR receptors (22). Furthermore, different KIR haplotypes—group A and B—have been identified. While the group A haplotype comprises almost exclusively inhibitory, the group B haplotypes also encode activating KIRs (23). Indeed, most receptors are expressed only on subsets of NK cells. Therefore, NK cells are not a uniform cell population but composed of many different subsets that differ in their mode of activation and their functional properties. In this review, we will discuss some of the NK cell subsets that have been studied in HCV and HCMV infections (Figure 1).

## NK Cell Subsets Carrying Specific KIRs

One receptor that has repeatedly been implicated in the anti-HCV immune response is the inhibitory KIR2DL3. In 2004, Khakoo and colleagues (24) reported a genetic association between the expression of KIR2DL3 and HCV clearance. They studied more than thousand patients infected with HCV, of whom 685 developed a chronic infection while 352 cleared the virus. Those patients, whose NK cells expressed the inhibitory KIR2DL3 homozygously on an HLA-C1/C1 background, were more likely to clear the virus spontaneously. Of note, when stratified in terms of route of infection, this association was only observed in patients with presumed low-dose viral inoculum, e.g., after a needle-stick, suggesting that the antiviral NK cell response is most efficient in situations with low viral load. Other studies in cohorts of injection drug users confirmed that exposed but uninfected individuals had a higher frequency of KIR2DL3 expression than drug users with chronic and resolved infections or healthy donors (25). This association was also reflected in the response to treatment, in which KIR2DL3–HLA-C1 expressing patients had a higher chance to achieve SVR after IFN-α-based treatment (26). However, in other studies, a correlation between KIR2DL3 and outcome of infection was not observed, albeit with smaller and different patient cohorts (27, 28). The current hypothesis for this association is that NK cells expressing KIR2DL3, which has a rather weak binding affinity to HLA-C (29), tend to receive less inhibitory signals and are, therefore, more easily activated than NK cells expressing KIRs with higher binding affinities.

Additionally, there is now increasing evidence that peptides presented in MHC class I complexes also influence binding affinities of KIRs. This has already been shown for HIV peptides (30–32) and could in 2016 for the first time be shown for an HCV peptide presented by an HLA-C1 molecule to KIR2DL3 on NK cells (33). Lunemann et al. (33) identified peptides derived from HCV NS3 and Core protein that stabilized expression of HLA-C*03:04 on transfected 721.221 cells and facilitated binding of KIR2DL3–Fc proteins. One of the peptides derived from HCV Core protein was furthermore able to inhibit degranulation of primary KIR2DL3^+^ NK cells. When comparing core peptide sequences from different HCV genotypes, the genotype 1-derived peptide provided the strongest inhibitory signal, while peptides from other genotypes were much less effective. These results encourage speculations about possible viral escape mechanisms by modulation of KIR binding and the implication that NK cells exert evolutionary pressure on the HCV genome. Future studies should address if the identified peptides can also be presented on primary hepatocytes and if other KIRs/HLA interactions are involved as well.

Moreover, there is substantial evidence for an overrepresentation of certain KIRs in expanded adaptive NK cell subpopulations (see below) in HCMV. The currently available data yield a complex picture with some reports highlighting KIR2DL2/3 (34–36) others KIR3DL1 (37) or activating KIRs (38).

## The CD56^neg^ NK Cell Subset

Traditionally, NK cells are classified as CD3^−^CD56^+^ lymphocytes, which are further divided into a CD56^dim^CD16^+^ and a CD56^bright^CD16^−^ subset. During chronic viral infections, especially in HIV and HCV, a subset of CD3^−^CD56^−^CD16^+^ NK cells is detectable (39). These cells miss expression of lineage markers, such as CD14 and CD19 or markers of other cell types positive for CD16, while expressing a variety of NK cell receptors (NCR, KIR, and NKG2). They are, therefore, classified as CD56^neg^ NK cells. This NK cell subset is found at low percentages (around 5% of all NK cells) in healthy adults and even in neonates, but can expand to 10–40% of all NK cells during HIV, acute, chronic, and resolved HCV infections or HIV/HCV coinfection. Concurrently, a drop in the percentage of CD56^dim^ NK cells is observed (40–42).

Phenotypically, CD56^neg^ NK cells from healthy donors and chronic HCV patients are similar and expression of many receptors is comparable between CD56^neg^ and CD56^dim^ NK cells from chronic HCV patients. Only CD57 and to a lesser extent NKp30 were found to be expressed at lower levels in the CD56^neg^ NK subset (40). Functionally, however, CD56^neg^ NK cells appear impaired. In response to different stimuli, the CD56^neg^ NK subset of HCV patients failed to secrete significant amounts of IFN-γ and TNF-α and displayed low perforin expression and degranulation (40, 42). Yet, they showed higher TRAIL expression compared to CD56^neg^ NK cells from healthy controls (41). This functional impairment seems to be a general feature of the CD56^neg^ NK cell subset, as it was observed in various chronic inflammatory situations. However, overnight *in vitro* culture of CD56^neg^ NK cells with IL-2, IL-12, or IL-15 resulted in cytotoxicity levels comparable to stimulated CD56^+^ NK cells, indicating that upon stimulation CD56^neg^ NK cells can effectively function (43). It was shown in HCV, as well as in HIV/HCV coinfection, that high pre-treatment levels of CD56^neg^ NK cells correlated with treatment failure (40, 44). Furthermore, after successful IFN-α/ribavirin treatment of HIV/HCV coinfected patients, absolute levels and percentage of CD56^neg^ NK cells normalized after 4 weeks (45).

So far, the role of this particular NK cell subset was not evaluated during DAA treatment of patients, but several lines of evidence suggest that the decline in CD56^neg^ NK cells after treatment is not induced by IFN-α, but results from a decrease in viral load (39). Until now, it was shown that the CD56^dim^ and CD56^bright^ NK cell subsets quickly normalize after DAA treatment in regard to numbers and functionality (46–48). This observation could indicate that the CD56^neg^ subset also normalizes. However, further studies are needed to address this and other open questions: does the CD56^neg^ subset represent a terminally differentiated or exhausted cell type or rather a specific lineage? What is the degree of plasticity in terms of other NK cell subsets becoming CD56^neg^ or CD56^neg^ NK cells acquiring CD56^pos^ phenotypes? The fact that neonates and healthy individuals already have this CD56^neg^ subset, might argue against an exhausted phenotype, although many of their properties resemble exhausted T cells.

In contrast to HCV and HIV, increased levels of CD56^neg^ cells have only been reported sporadically in HCMV infection and were observed only in a subset of patients experiencing viral reactivation (49).

## The NKp46^high^ NK Cell Subset

Expression of NK cell receptors was analyzed in different HCV patient cohorts (e.g., in acute or chronic infection and during treatment), but the obtained results have been highly controversial (50, 51). With regards to NKp46, however, several independent studies observed the involvement of an NKp46^high^ NK cell subset expressing multiple markers of immature NK cells (52) in protection from infection, spontaneous clearance, liver inflammation, progression of fibrosis, and outcome of treatment.

In prospective studies of injection drug users, a higher percentage of CD56^dim^ NK cells with increased levels of NKp46 was correlated with a higher percentage of individuals remaining seronegative, suggesting that high expression of NKp46 might be predictive for protection from infection (25). However, Alter et al. (42) reported lower expression of activating receptors, including NKp46, in patients with acute infection who subsequently cleared the virus than in those, who progressed to chronic infection.

In chronically infected patients, the majority of studies report elevated NKp46 expression on peripheral blood NK cells compared to healthy donors (51). Of note, this is even more pronounced in the liver (52). NKp46^high^ peripheral NK cells from healthy donors and HCV patients perform better in reducing HCV-RNA from *in vitro* infected hepatocytes, produce higher levels of IFN-γ, and degranulate more *ex vivo* in response to different stimuli (52, 53). Likewise, intrahepatic NKp46^high^ NK cells from HCV patients were shown to degranulate more *ex vivo* than NKp46^dim^ cells (54). Furthermore, staining with an NKp46-Ig fusion protein revealed higher expression of a yet unknown NKp46-ligand on HCV-infected Huh7.5 hepatoma cells than on uninfected cells (53).

Accordingly, NKp46 levels in patients correlate positively with liver inflammation scores (54) and inversely with HCV serum levels (52), suggesting that NKp46^high^ NK cells can kill infected hepatocytes and contribute to viral control during chronic infection. The NKp46^high^ subset correlated with low fibrosis stages, possibly due to NKp46-dependent killing of hepatic stellate cells, the main drivers of fibrosis (54–56).

Even though the NKp46^high^ NK cell subset might be beneficial in reducing viral load and liver fibrosis during chronic infection, it also predicts failure to IFN-α therapy (54, 57). After successful DAA treatment, previously elevated NKp46 levels in liver and blood normalize, concomitantly with a normalization of many other NK cell receptors (47).

In contrast, in HCMV infection, there is little evidence for a direct modulation or involvement of NCRs. One report observed the dissociation of the CD3zeta chain from NKp30 after engaging the HCMV tegument protein pp65, leading to greatly reduced NKp30-mediated killing (58).

## Adaptive NK Cell Subsets

The most striking example for a long-lasting impact of a pathogenic challenge on distinct NK subsets (59) was initially identified in two key reports by Miguel Lopez-Botet’s group (60, 61). In HCMV-seropositive individuals, a higher proportion of NK cells expressing the activating receptor CD94/NKG2C was detected. This expanded subset displays lower NCR levels and increased expression of CD85j/LIR-1 (60) and CD2 (38). Similar observations were made in transplant recipients who suffered from acute CMV infection/reactivation (34, 37, 49, 62, 63). *In vitro* studies recapitulated subset expansion suggesting that exposure of NKG2C^+^ NK cells to infected cells was critical for this process (61). Moreover, the interaction between HLA-E and CD94/NKG2C was defined as a critical event for subset expansion (64, 65). To date, several additional factors have been reported to contribute to the expansion and activation of NKG2C^+^ NK cells in response to HCMV infection, such as IL-15 (61), IL-12 (64), and CD14^+^ monocytes (64, 66), as well as the interaction between CD2 and upregulated CD58 on infected cells (67). As expansion of subpopulations and their subsequent longevity resemble hallmarks of adaptive immune responses, the term “adaptive NK cells” was coined for human NK cells displaying these characteristics. From here on, we will use the term in this broadly defined sense, comprising multiple subsets.

Intriguingly, while a large number of studies describe NK subset expansions in other infections, e.g., HIV (68–70) Hantavirus (65), Chikungunya virus (71), EBV (72), and HBV/HCV (35, 73), seropositivity for HCMV seems to be a necessary pre-requirement. Altered HLA-E levels and/or a certain inflammatory cytokine milieu could be common denominators permitting the (re)expansion of NK cells “primed” initially by HCMV. The initial events, however, underlying the formation of this NK cell subpopulation in primary HCMV infection, remain enigmatic and represent a field of intense interest.

In recent years, it became clear that the initial definition as NKG2C^+^ (and CD57^+^) was not sufficient to encompass all adaptive NK cell subsets.

A study by Hwang and coworkers in 2012 (74) identified NK cell subpopulations with low or absent expression of the adaptor protein FcεRIγ in about one-third of all individuals tested. Zhang et al. (75) then established that the presence of FcεRIγ-deficient NK cells was strictly associated with prior exposure to HCMV. Expansion of an FcεRIγ^−^ subset was also observed in HCMV^+^ chronic HCV patients and correlated with low liver damage and fibrosis levels, possibly implying an involvement of this subset in protection from immunopathology (76). Further reports extended the concept of HCMV driving the expansion of adaptive NK cell populations with deficiencies in key signaling molecules to Syk, EAT-2, and DAB2 and the transcription factors PLZF and Helios (77, 78). These features are not necessarily combined at a single-cell level and instead found in different combinations creating a previously unappreciated heterogeneity among adaptive NK cells.

Intriguingly, this molecular signature partially resembles exhausted T cells and in fact a recent study described high PD-1 expression on a subset of CD57^+^ NK cells also displaying increased LIR-1 levels as well as higher NKG2C expression in some donors (79). Together with lower NCR expression in adaptive NK subsets, these features suggest a decreased functionality. While this seems to be the case for classical tumor targets (78), superior antibody-dependent cellular cytotoxicity (ADCC) responses are emerging as a prominent and distinct characteristic of adaptive NK cells (38, 75, 77, 80–82), augmented by CD2 co-stimulation (67, 83). This functional specialization is accompanied by broad epigenetic modifications, including better accessibility of the *ifn-*γ locus (77, 78, 84). Latent HCMV infection might, therefore, be a worthy trade-off for the host if the interplay of CMV-induced adaptive NK cell populations and antigen-specific humoral immunity *via* ADCC results in elevated resistance to heterologous infection.

Besides deciphering the generation and function of adaptive NK cell subsets, several studies focused on their localization. In mice, a subset of NK cells endowed with antigen-specific memory has been shown to reside specifically in the liver (85, 86). In human liver samples, a subset, phenotypically similar to memory NK cells in the mouse (CD49a^+^T-bet^+^Eomes^−^) that also displayed high NKG2C expression, was identified. Yet, in contrast to peripheral NKG2C^+^ NK cells, they had an immature phenotype (CD57^−^, CD16^−^CD56^bright^), existed in high numbers in HCMV negative donors, and were incapable of mounting ADCC responses due to lack of CD16 expression (85, 87). Therefore, besides their striking resemblance to murine liver-resident memory NK cells and some shared features with human peripheral blood adaptive NK cells, the function of this unique human liver NK subset needs to be further defined.

Very recently, another liver-resident NK cell population, characterized as Eomes^hi^, CXCR6^+^ (88–90), and CD49e^−^ (91) was described, following up HLA-mismatched human liver-transplants. The authors demonstrated that these Eomes^hi^ NK cells survive for up to 13 years (90). This remarkable longevity makes an involvement of this subset in tissue homeostasis or antiviral responses against chronic infections a plausible scenario, which awaits further investigation.

## Conclusion

Virus infections, especially with persistent viruses, have a remarkable impact on the NK cell compartment, shape the overall NK cell repertoire, and profoundly affect their effector functions. However, *vice versa*, the different NK subset composition and receptor distribution before infection can also be decisive how well infections can be combated. The diversity of human NK cell subsets is one of the emerging topics in the field. Especially, tissue-resident NK cells and other subsets of helper innate lymphoid cells (ILCs), their development, regulation, antiviral functions, and plasticity in tissues, such as in the liver, are currently an area of intense research. A better understanding of the development and dynamics of ILCs comprising both NK cells and helper ILCs subsets in affected tissues during chronic viral infection might help the design of improved targeted strategies for therapeutic intervention.

## Author Contributions

JP and AR wrote the manuscript. MH revised the manuscript. AC revised the manuscript and provided conceptual input.

## Conflict of Interest Statement

The authors declare that the research was conducted in the absence of any commercial or financial relationships that could be construed as a potential conflict of interest. The reviewer, RJ, and handling editor declared their shared affiliation, and the handling editor states that the process, nevertheless, met the standards of a fair and objective review.
